# Toolkits to Support Health Care Providers in Health Behavior Change for Patients With Chronic Conditions: Scoping Review

**DOI:** 10.2196/88653

**Published:** 2026-10-26

**Authors:** Jillian Scandiffio, Robert Simpson, Ashvene Sureshkumar, Jerry Tang, Paloma Fleming, Dina Gaid, Harzaan Gnanakaran, Marina Wasilewski, Nicole Woods, Sarah J Donkers, Sarah Munce

**Affiliations:** 1 Rehabilitation Sciences Institute University of Toronto Toronto, ON Canada; 2 KITE Research Institute Toronto Rehabilitation Institute University Health Network Toronto, ON Canada; 3 St. Michael's Hospital Unity Health Toronto Toronto, ON Canada; 4 Faculty of Medicine University of Ottawa Ottawa, ON Canada; 5 Schulich School of Medicine & Dentistry Western University London, ON Canada; 6 Faculty of Science McMaster University Hamilton, ON Canada; 7 St. John's Rehab Research Program Sunnybrook Health Science Centre Toronto, ON Canada; 8 The Institute for Education Research University Health Network Toronto, ON Canada; 9 Institute of Health Policy, Management, and Evaluation University of Toronto Toronto, ON Canada; 10 School of Rehabilitation Science University of Saskatchewan Toronto, SK Canada; 11 Bloorview Research Institute Holland Bloorview Kids Rehabilitation Hospital Toronto, ON Canada

**Keywords:** toolkit, health behavior, development, design, knowledge translation, scoping review

## Abstract

**Background:**

Health care providers (HCPs) are key agents in supporting health behavior (eg, smoking and physical activity) change in patients. Toolkits have previously been used to guide HCPs in care and have shown effectiveness in improving clinical outcomes. However, there is a lack of understanding of how toolkits should be developed to best support HCPs.

**Objective:**

This scoping review aimed to map the existing literature on the theories, models, frameworks, approaches, and methods that have been used in the development of toolkits to support HCPs in health behavior change.

**Methods:**

Five databases (MEDLINE, Embase, CINAHL, Web of Science, and Scopus) were searched from inception to August 2026. Original research articles published in peer-reviewed academic journals in English from any geographic area describing the development of a toolkit to support HCPs in health behavior change for patients with chronic conditions were included. Qualitative data were analyzed using conventional content analysis and quantitative data were examined in tabular terms, with a simple descriptive synthesis of the findings. Barriers and facilitators identified from studies were deductively coded using the Theoretical Domains Framework (TDF) and reported according to TDF domains identified in studies.

**Results:**

Nineteen articles met inclusion criteria. The articles included toolkits addressing several health behaviors, including physical activity (n=16), nutrition (n=6), sedentary behavior (n=2), sleep (n=2), and weight management more broadly (n=2). Toolkit formats (eg, use of videos and written components) varied greatly between studies. All studies included knowledge users in the development of the toolkit. Use of a theory, model, or framework was described in 14 studies, and barriers and facilitators to HCP promotion of health behavior change were examined in 14 studies. The most frequently reported TDF domain was environmental context and resources, with time as the most frequently cited barrier. Toolkit development typically consisted of an exploratory phase to determine knowledge users’ needs and wants, toolkit development, dissemination, and evaluation.

**Conclusions:**

Toolkits varied greatly in format and development process but generally involved iterative development based on feedback from knowledge users. However, no toolkits targeted smoking, alcohol use, or recreational drug use, which warrant further study. To assist future investigators looking to develop a toolkit for health behavior change, we present a definition of a toolkit and a series of recommendations for toolkit design and development. Although previous reviews have mapped the existence of various toolkits, they have not examined how these have been developed. This review provides a novel description of this development and a guide for the development of future toolkits to increase uptake of toolkits and reduce the knowledge-to-practice gap. Additionally, this review defines the term “toolkit” and identifies its minimum components, which can be used by researchers to classify and compare toolkits in the future.

**Trial Registration:**

OSF Registries osf.io/bstmy; https://osf.io/bstmy/overview

## Introduction

### Rationale

Lifestyle behaviors (eg, smoking, physical activity, and nutrition) are indicators of population health more broadly [1-4] and are associated with a range of health outcomes, including comorbidity (eg, cardiovascular disease, diabetes, and cancer) [5,6], health service use [7-9], and quality of life [10]. These behaviors are multifactorial and include a series of behaviors addressing both physical and psychosocial health [11]. Health care providers (HCPs) are key agents in supporting health behavior change in a variety of clinical populations [12-14]. However, there is evidence to suggest that HCPs’ ability to address health behaviors in day-to-day care is hindered by multiple external factors, including, but not limited to, lack of confidence in counseling patients about behavior change, lack of time to discuss behavior change, and health system gaps [15].

It is important to develop user-friendly, evidence-based tools that support HCPs in promoting health behavior change in their patients. Toolkits are collections of adaptable resources to inform and provide guidance that are typically intended to bridge the evidence-to-practice gap, support implementation and adoption, and/or support sustained use of the intended change [16]. The definition of what encompasses a “toolkit” is somewhat heterogeneous, as toolkits range greatly in modality, formatting, and content and may include individual documents or combinations of documents such as timelines, educational materials, and assessment instruments [16,17]. Toolkits can be used by themselves as a knowledge mobilization strategy or can be part of a larger multistrategy intervention [17]. Toolkits have shown effectiveness in improving clinical outcomes (eg, reduced patient falls, decreased hospital length of stay, and improved diabetes management) in a range of patient populations (eg, cancer, diabetes, and depression) [17-19]. The wide range of toolkit designs suggests that, before a toolkit is developed and implemented, there should be a clear understanding of how it should be developed. However, there has been limited evidence on how toolkits have been developed in this context. An evidence-informed approach to toolkit design may improve toolkit engagement, uptake, and effectiveness.

While scoping reviews have been conducted on toolkits in health care [17,20], there is a lack of information regarding the design and development of these toolkits. When referring to design, this includes considerations of what the toolkit should look like and its components, including how the modality of the toolkit will be used and understood by end users. When referring to development, this includes considerations of the process of creating the toolkit, including stages such as identifying knowledge gaps, adapting the toolkit to local needs, and implementing the toolkit. In short, when we refer to design, we are referring to the what (the toolkit) and when we refer to development, we are referring to the how (the process of creating the toolkit). Additionally, there have been recommendations for development of a toolkit in areas outside of health behavior change (eg, quality improvement) [21], but there is a lack of information regarding best practices for the development of toolkits to support HCPs in health behavior change. Further, there is a lack of information regarding best practices for the development of toolkits to support HCPs in health behavior change. For example, a recent review examined toolkits for physical activity adherence in patients with chronic conditions and noted that the process of toolkit development was not specified in any of the included studies [22]. This includes a lack of information about theories, models, or frameworks (TMFs) that guide toolkit development. It is important to consider how and whether TMFs have been used in toolkit development, as understanding the theoretical approaches used can enhance understanding of why implementation succeeds or fails [23]. Further, using TMFs is crucial for enabling knowledge translation and implementation of interventions into practice [24]. Thus, there is a gap in understanding best practices for the development of a toolkit to support HCPs in addressing health behavior change, which this review aimed to address. By gaining a deeper understanding of the optimal approaches to toolkit development, we can better design future toolkits for HCPs. The findings from this review may act as a resource for investigators looking to develop future toolkits for HCPs in health behavior change and more broadly.

### Objective

The objective of this scoping review was to map the existing literature on the TMFs, approaches, and methods used in the development of toolkits to support HCPs in health behavior change.

## Methods

### Protocol and Registration

This review was undertaken in accordance with the Joanna Briggs Institute (JBI) methodological guidance for conducting scoping reviews [25]. The protocol was registered with the Open Science Framework on November 22, 2024, and published [26]. There were no deviations from the registered protocol. The reporting of the review follows the PRISMA-ScR (Preferred Reporting Items for Systematic Reviews and Meta-Analyses Extension for Scoping Reviews) guidelines (Multimedia Appendix 1) [27].

### Eligibility Criteria

#### Overview

Studies were included in the review based on population, concept, and context (PCC) criteria [25].

#### Types of Participants

When considering population, studies were included that involved a toolkit targeted to HCPs for changing the health behaviour of patients with chronic conditions. Chronic conditions were defined as “conditions that last 1 year or more and require ongoing medical attention or limit activities of daily living or both” [28]. HCPs needed to be identified as end users or the target audience for the toolkit for the study to be included. If a toolkit was meant for use by another end user (eg, patients) and did not note that HCPs were also intended end users, the study was not included.

#### Concept

The conceptual focus was on the approach to developing toolkits to support HCPs in addressing lifestyle-related health behavior change among their patients with chronic conditions. Health behaviors were categorized according to the categories reported by Statistics Canada [1], including negative health behaviors (smoking or tobacco use, recreational drug use, and alcohol use), movement behaviors (eg, physical activity and sedentary behavior), sleep, and nutrition. Chronic conditions were based on those identified by Statistics Canada and the Public Health Agency of Canada [29,30], with additional chronic conditions included when relevant (eg, a rare chronic neurological condition not explicitly mentioned by the organizations but falling within a broader category of “neurological condition”). Studies were included if they (1) described the development of a toolkit to support HCPs in health behavior change and (2) had a primary focus on the toolkit development approach. Studies in which authors described their intervention as a “tool” or a related term (eg, toolkit and toolbox) were included, but studies where the intervention was not referred to using these terms (eg, package and materials) were excluded.

#### Context

Included studies were limited to those reporting original research that were published in English in peer-reviewed academic journals.

#### Types of Evidence Sources

All study designs were considered, provided that they reported primary research data and described the approaches for developing a toolkit.

### Information Sources

The search is reported according to the PRISMA-S (PRISMA Search Reporting Extension) [31]. The search strategy was developed in collaboration with an academic librarian and a Peer Review of Electronic Search Strategies (PRESS) [32] was conducted by a second academic librarian. The search was first conducted in MEDLINE (Ovid) and then was translated to the following additional databases: Embase (Ovid), CINAHL (EBSCOhost), Web of Science (Clarivate), and Scopus. Each database was searched separately. The reference lists of all included sources of evidence were screened for additional studies. No additional online or print sources were purposefully searched (eg, table of contents, websites). We searched each database from inception onward, with the initial search conducted in November 2024 and the search was rerun in August 2026. No limits were used in the search. No additional studies or data were sought by contacting others (eg, authors). The search strategy was neither adapted nor reused from other literature reviews. Full search details are available in Multimedia Appendix 2.

### Selection of Sources of Evidence

Search results were imported, deduplicated, and screened in Covidence (Veritas Health Innovation). Each title/abstract was reviewed against the inclusion criteria for eligibility by 2 independent reviewers (JS, AS, JT, PF, DG, or HG), with the full texts of potentially eligible studies reviewed in by 2 independent reviewers (JS, AS, JT, PF, DG, or HG) against inclusion criteria. If disagreements occurred, consensus was reached through discussion, with a third reviewer (SM) available for arbitration. To ensure reliability and consistency in screening, a pilot review of 100 of the titles was screened by reviewers to establish interrater reliability, with a Cohen κ of 0.8 as the threshold raters needed to meet, indicating substantial agreement [33].

### Data Charting Process

Data were extracted from included articles by 2 independent reviewers using a data extraction tool developed by the research team, guided by the JBI template (Multimedia Appendix 3) [34]. The data extraction tool was developed and piloted at the protocol stage and trialed in the first few studies by 2 independent reviewers to ensure that all relevant results were extracted. Any disagreements that arose between reviewers were resolved through discussion, or by consultation with an additional senior reviewer.

### Data Items

Data charted included authors, year of publication, origin/country of publication, country of toolkit development, study aims/purpose, HCP population (eg, nurses and allied health professionals), patient population of interest, PROGRESS+ considerations [35] in toolkit tailoring (eg, gender and race/ethnicity), health behavior, toolkit aim, toolkit componentry, toolkit development, evaluation process, TMFs applied, methods, approaches, knowledge users involved, outcomes, and authors’ recommendations/lessons learned. For this review, a theory is defined as “a set of analytical principles or statements designed to structure our observation, understanding, and explanation of the world,” a model is defined as a simplification or representation of a specific aspect of a phenomenon, and a framework as an explanation of a phenomenon through a structure, overview, outline, system, or plan of descriptive categories and the relationships between them [23].

### Synthesis of Results

Conventional content analysis [36] was undertaken for qualitative data, with a simple descriptive synthesis of the findings. Quantitative data were examined in tabular terms and reported descriptively. Barriers and facilitators identified from studies were deductively coded using the Theoretical Domains Framework (TDF) [37] and reported according to the TDF domains identified in the studies. This framework was selected because it considers behavior change theories and was originally developed to identify influences on HCP behavior for implementing evidence-based recommendations [38]. A series of recommendations was developed regarding toolkit design and development based on the barriers and facilitators and recommendations described in included studies. The most commonly reported barriers, facilitators, and recommendations across studies were presented to an integrated knowledge translation panel, including people with chronic conditions, implementation scientists, HCPs, and researchers with expertise in health behaviors. Final recommendations for design and development were developed through iterative discussions with panel members, where members agreed on recommendations that should be included and provided feedback on their wording.

## Results

### Selection of Sources of Evidence

Our database search identified 24,658 studies for possible inclusion (Figure 1). Following deduplication, a total of 20,978 studies were screened for inclusion at the title and abstract stage, and a subsequent 120 full texts were examined. After screening, 19 studies were included in the scoping review [39-57].

**Figure 1:**
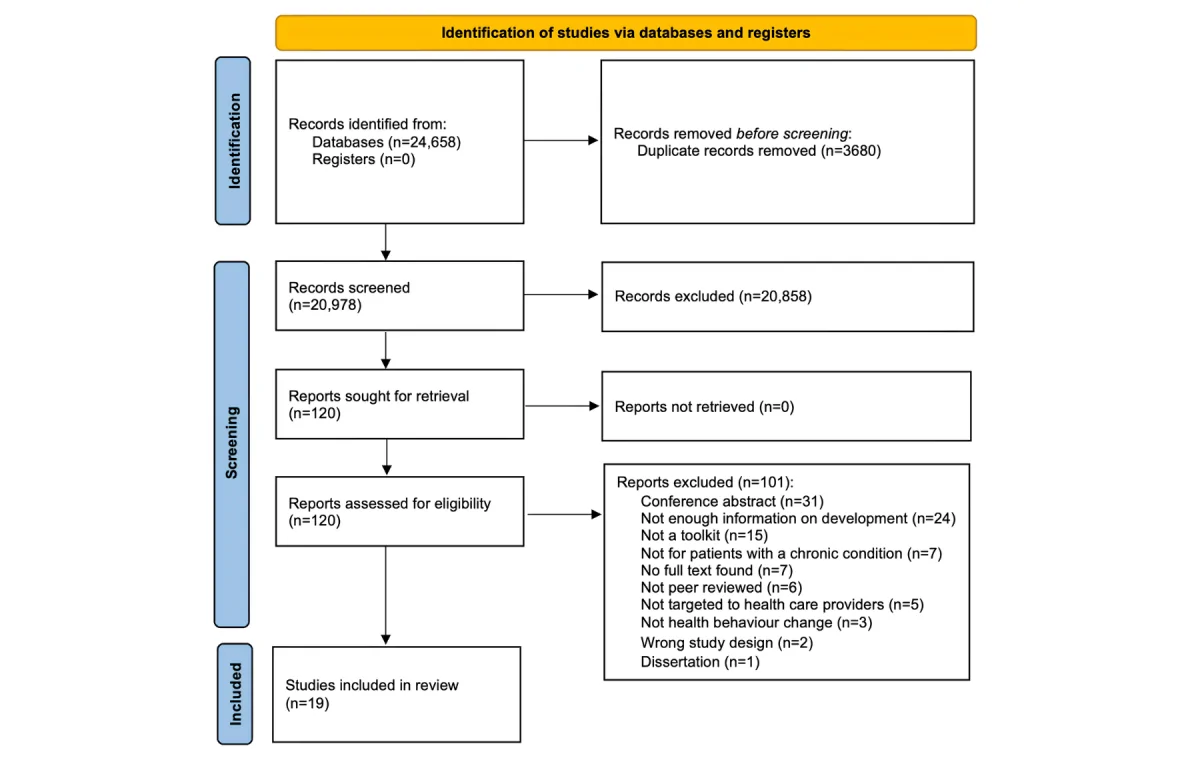
PRISMA (Preferred Reporting Items for Systematic Reviews and Meta-Analyses) flow diagram of included studies.

### Characteristics of Sources of Evidence

Included studies were published between 2007 and 2025 (Multimedia Appendix 4). Studies took place in the following countries: Canada (n=7) [39-42,45,47,48], Australia (n=5) [42,49,51,52,57], the United Kingdom (n=4) [43,44,50,54], Netherlands (n=2) [46,56], and the United States (n=2) [53,55]. It should be noted that one of these studies took place in both Canada and Australia [42]. Approximately half of the studies (n=10) [40-43,45,47,48,51,54,56] used mixed or multiple methods and approximately half (n=8) [39,44,46,49,50,52,53,55] used qualitative methods for toolkit development; no studies used only quantitative methods.

### Characteristics of Included Toolkits

When classifying the toolkit, 13 studies used the term “toolkit” when referring to their intervention [39-42,45,47-51,53,54,57], 1 study used the term “toolbox” [52], and 5 studies referred to it only as a “tool” [43,44,46,55,56]. Only one study provided a citation for the concept of a toolkit [57]. Toolkit modalities varied, including web-based (n=8) [42,45,48-51,55,56], hard-copy (n=3) [44,53,54], and hybrid (n=4) [41,46,47,57] formats. Toolkit modality was unclear or not specified in 4 studies [39,40,43,52]. The toolkits were used in combination with other activities (eg, training program) in 4 studies [53-56] and was the sole intervention in 15 studies [39-52,57]. Selected toolkit characteristics are visually represented in Figure 2.

**Figure 2:**
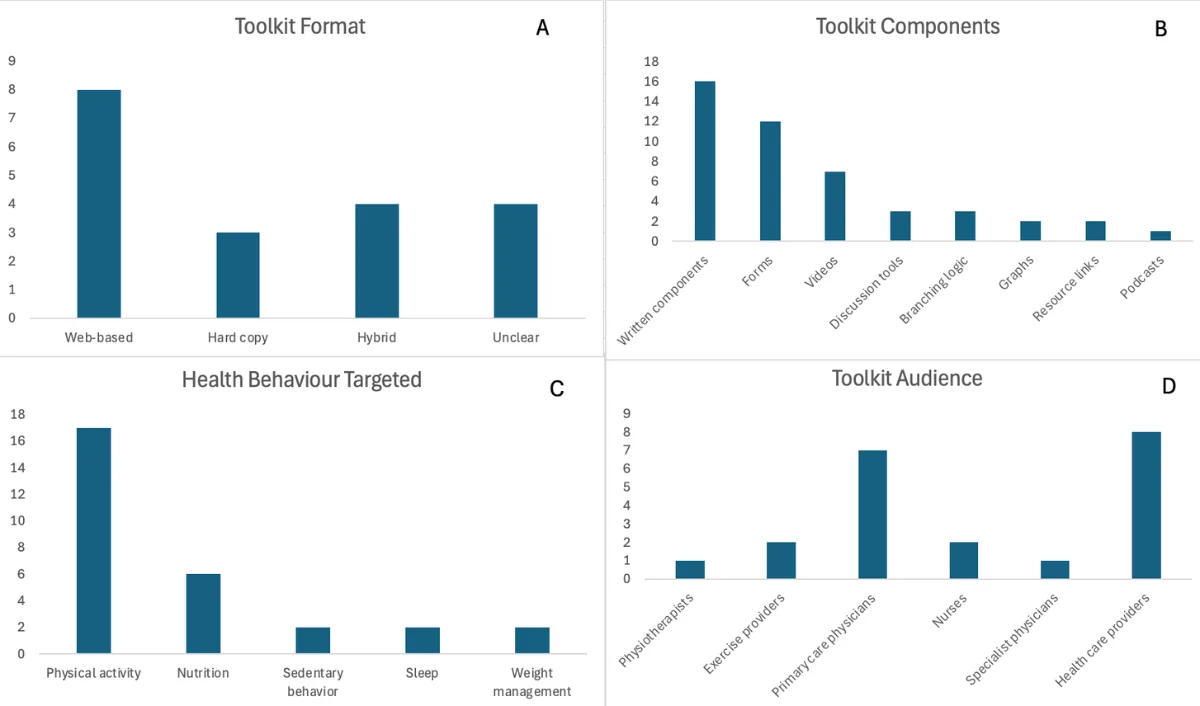
Frequency of the reported characteristics of included toolkits, including (A) toolkit format, (B) toolkit components, (C) the health behavior targeted by the toolkit, and (D) the toolkit’s target audience.

Toolkit components varied greatly between studies. Five studies [39,41,49,50,52] used only one type of component (eg, written information only or forms only), while the other 14 toolkits [40,42-48,51,53-57] were multimodal. Written components such as summaries of evidence were included in 16 toolkits [40,42-55,57]. Further, 12 toolkits included forms for patients and/or HCPs to complete together (eg, SMART forms) [39-44,48,53-57]. Additional toolkit components included videos (n=7) [42,45-48,51,54], discussion tools (eg, conversation dice; n=3) [46,51,53], branching logic to make recommendations on care (n=3) [51,55,57], graphs (n=2) [40,53], links to resources (n=2) [43,56], and podcasts (n=1) [42].

The health behaviors targeted by the toolkit included physical activity/exercise (n=17) [40-52,54-57], nutrition (n=6) [40,41,49-51,55], sedentary behavior (n=2) [41,56], sleep (n=2) [40,41], and unspecified weight management (n=2) [39,53]. Approximately half of the toolkits addressed only one health behavior (physical activity) [42-48,52,54,57] and the other half addressed multiple health behaviors [39-41,49-51,53,55,56]. The HCP audience for the toolkits included physiotherapists (n=1) [46], exercise providers (n=2) [42,45], primary care physicians (n=7) [40,49,50,53-56], nurses (n=2) [50,56], specialist physicians (n=1; cancer care workers) [43], and HCPs more broadly (n=8) [39,41,44,47,48,51,52,57]. The toolkits were targeted to a range of patient populations, including obesity (n=6) [39-41,49,53,55], neurological conditions (n=4) [44,45,52,57], cancer (n=2) [42,43], diabetes/prediabetes (n=2) [50,54], physical disability (n=2) [46,47], depression (n=1) [48], osteoarthritis (n=1) [51], and patients with both type 2 diabetes and chronic obstructive pulmonary disease (n=1) [56].

### Toolkit Development Process

All studies reported including knowledge users in the development process. Most studies included HCPs (n=18) [39-51,53-57] and patients (n=17) [39,41-52,54-57], with additional knowledge users including subject experts/researchers (n=8) [44-48,50,51,56], community representatives (n=5) [45-49], care partners (n=5) [44-46,53,57], toolkit design specialists (n=3) [39,46,51], and implementation specialists (n=2) [47,48]. Participatory methods were reported in most studies (n=16) [39-48,50-52,54,56,57], including Co-X approaches (ie, co-design, co-development, co-creation, and co-production) methods (n=13) [39-46,50-52,54,57], integrated knowledge translation (n=2) [48,57], and participatory action research (n=3) [45-47]. Roles of knowledge users engaged in participatory methods included informing toolkit content or layout (eg, recommending topics the toolkit should cover and suggesting toolkit presentation; n=13) [39-47,50-52,54], study design (n=2) [41,45], assisting in study procedures (eg, recruitment and analyzing data; n=3) [45,50,56], developing toolkit materials (n=4) [39-41,47], reviewing toolkit prototypes (n=13) [39-48,51,52,57], and dissemination (n=5) [41,43,47,48,52]. Knowledge users were identified from HCP networks (n=8) [40,42-44,49,53,54,57], patient advisory organizations (n=5) [44,45,51-53], professional networks (n=4) [49,51,56,57], social media (n=3) [42,51,57], previous work (n=3) [51,55,57], community organizations/clinics (n=3) [45,52,57], and HCP grand rounds (n=1) [49]. Figure 3 provides a visual representation of the frequency of reported toolkit development processes.

**Figure 3:**
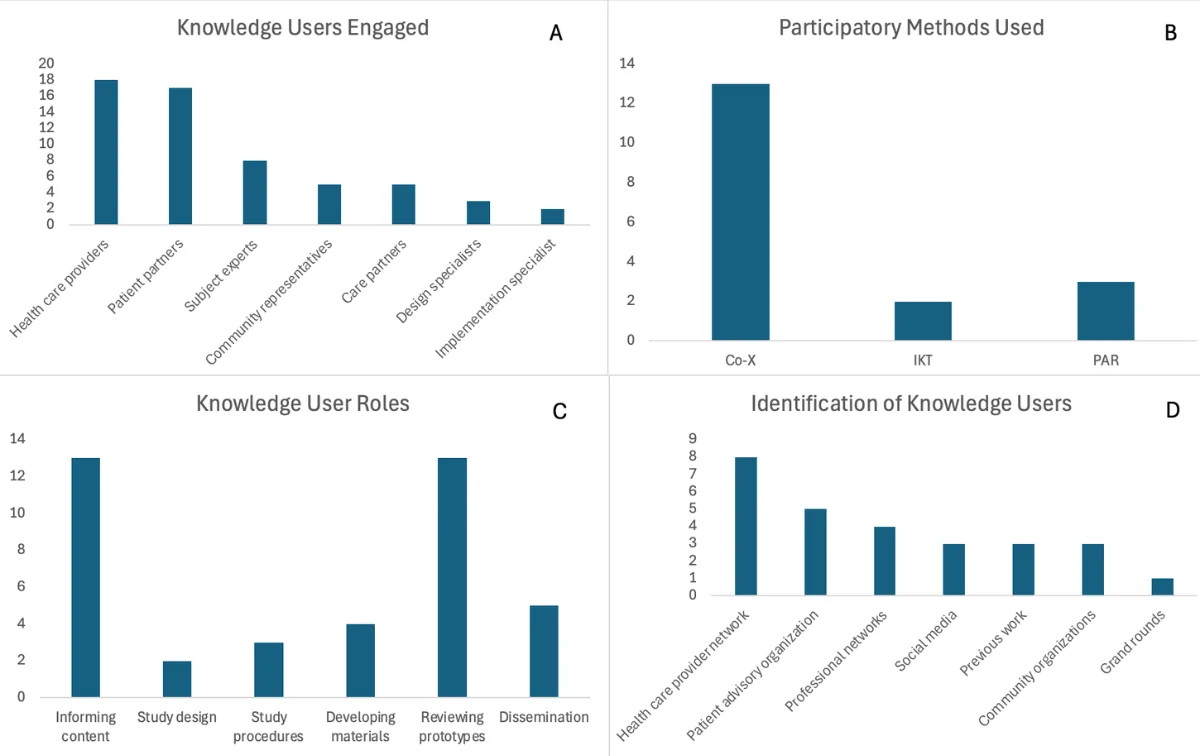
Frequency of the reported toolkit development process, including (A) knowledge users engaged, (B) participatory methods used in toolkit development, (C) roles of knowledge users engaged in participatory methods, and (D) how knowledge users included in participatory roles were identified. IKT: integrated knowledge translation; PAR: participatory action research.

### Theories, Models, and Frameworks

Use of TMFs in toolkit development was described in 15 studies [40-45,48-52,54-57]. These TMFs were from a range of disciplines, including education, psychology, and implementation science. TMFs used in multiple studies were the TDF [37] (n=5; [42,43,48,54,57]), Theory of Planned Behavior [58] (n=3; [54-56]), Knowledge-to-Action framework [59] (n=2; [45,49]), Normalization Process Theory [60] (n=2; [44,49]), Social Cognitive Theory [61] (n=2; [41,54]), the COM-B (capability, opportunity, and motivation–behavior) model [62] (n=2; [43,57]), and Self-Determination Theory [63] (n=2; [51,56]). More than one TMF was used in 11 studies [41,43,45,48-51,54-57], while 4 used only one TMF [40,42,44,52]. Operationalization of TMFs included informing toolkit content and layout (n=9) [40,41,48,50-52,54-56], analyzing results (n=6) [43,45,48,49,54,57], guiding the toolkit development process (n=4) [45,49,54,55], guiding questions asked to knowledge users (n=4) [42,44,54,57], informing intervention strategies (n=2) [45,48], and guiding knowledge user engagement (n=1) [45]. These findings can be seen in graphical format in Figure 4.

**Figure 4:**
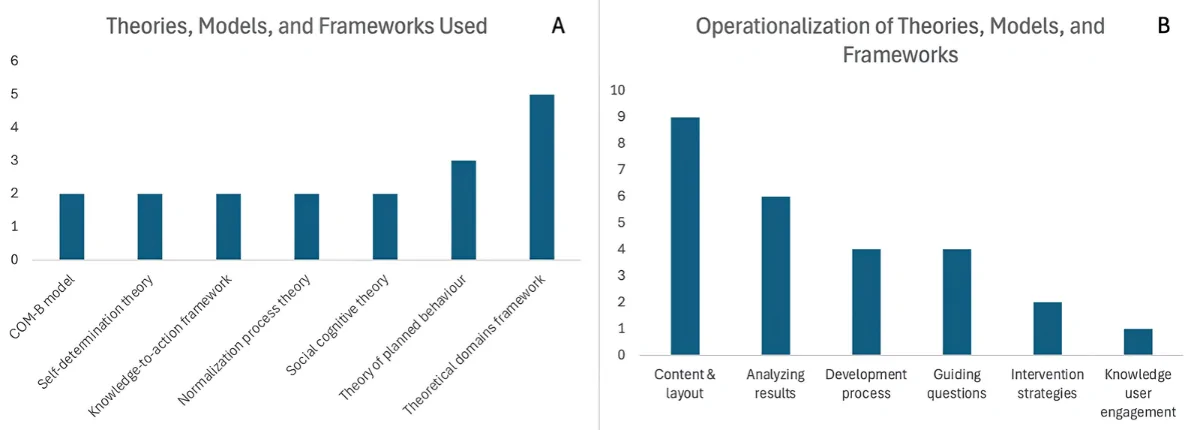
Frequency of the reported (A) theories, models, and frameworks used in multiple articles, and (B) how theories, models, and frameworks were operationalized. COM-B: capability, opportunity, and motivation–behavior.

### Phases of Development

Studies included 2-5 development phases. Development typically took the following phases: understanding the problem and doing preliminary work, toolkit development, dissemination, and evaluation. These phases, along with a description of some activities that occurred during each phase, are shown in Figure 5 and described further below.

**Figure 5:**
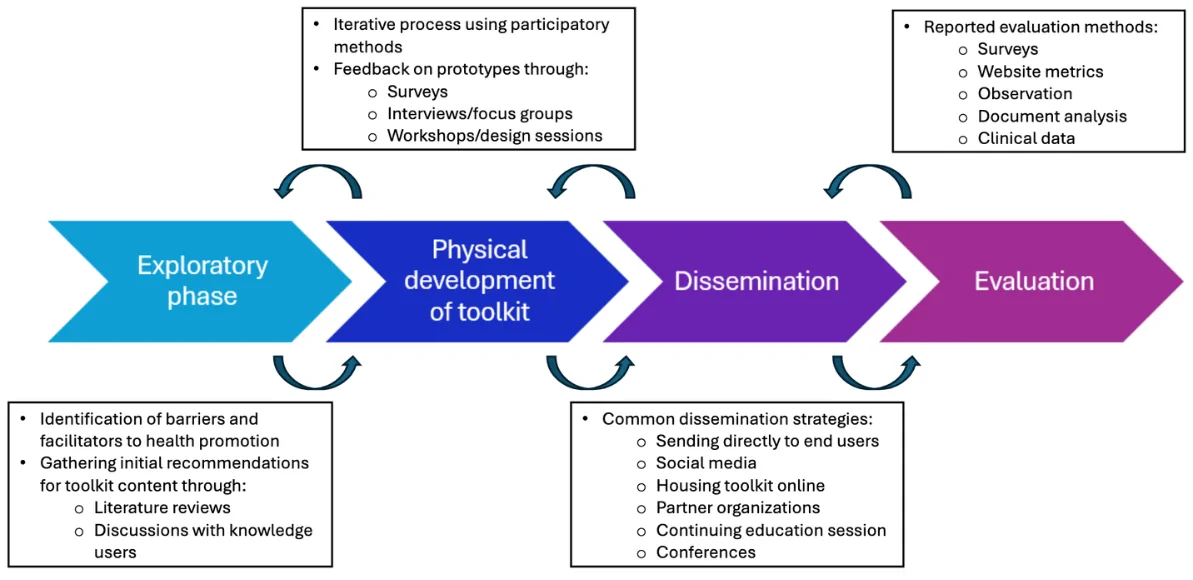
Phases of toolkit development seen in included studies, including information on activities seen during each phase.

During the exploratory stage, studies often used literature reviews and/or discussions with knowledge users to identify barriers and facilitators to health promotion behaviors and needs for an intervention to address a gap. In addition to gathering barriers and facilitators, this preliminary work also included literature reviews and/or discussions with knowledge users to gather recommendations for toolkit content (eg, what topics should be included, how the toolkit should be presented). Once this information was gathered, teams typically moved into the next phase of developing the toolkit itself.

The toolkit development phase was often an iterative process rather than a linear or a one-time process. Iterative practices often included discussions with knowledge users to get feedback; the number of times that these users were engaged throughout the process varied between studies. Methods of engaging knowledge users for feedback included surveys [40,41,43,45,47,51,54,57], interviews/focus groups [39-57], and workshops/design sessions [42,45,51,52,54,57]. Almost all studies (n=18) gathered recommendations from knowledge users and each of these studies incorporated these recommendations into the intervention [39-47,49-57].

Recommendations from knowledge users during the iterative design stage often included suggestions regarding language (ie, simplifying language and avoiding stigmatized terminology/using gentler language) [41,45,53,55,57], presentation (eg, readable font and use of color) [41,43,45,47,49,51,52,57], content additions (eg, increased information on exercise recommendations for specific groups) [40,43,45-47,49-51,53,54,57], and key messages [40,43,47,50]. Further, knowledge users provided feedback on structure [47,50-52,54,57], name suggestions [42,53], target audiences [45,52,56], dissemination strategies [47,50], and evaluation strategies [47]. We have provided a series of suggestions for the design of future toolkits based on commonly reported recommendations by study authors (Table 1). All studies incorporated recommendations from knowledge users, where appropriate, during iterative development of the toolkit.

**Table 1:** Recommendations for the design of future toolkits to support health care providers in health behavior change for patients with chronic conditions.

Recommendation	Rationale	Supporting evidence
Toolkits should include simple messages and avoid too much information (eg, including a summary sheet)	Time was cited as a frequent barrier to HCP^a^ promotion, so tools that are easy to reference and quick to use are needed	[40,42,47,49-51,57]
Toolkits should use lay terms and avoid jargon	Toolkits need to be easily accessible to and not confusing for end users, who may not be aware of technical terms	[41,45,55,57]
Toolkits should be visually appealing and use color, inclusive font, and images	Knowledge users highlighted the importance of making the toolkit visually appealing and easy to read to engage users	[41,43,45,47,49,52,57]
Toolkits should include practical examples and be easy for use	Including practical examples allows users to better understand and actively use the toolkit in practice	[39,40,42,45,47,53,57]
Toolkit information should be relevant to the patient population and tailored to their specific needs	End users recommended tailoring toolkit information to the patient population they are working with so that they can better provide recommendations based on the patients’ unique needs	[40,43,45,49,50,56,57]
Avoid sensitive terminology and harsh wording	Some terms that the research teams used were identified as charged/offensive by knowledge users who reviewed findings	[41,53,55]

^a^HCP: health care provider.

Dissemination strategies were described in 10 studies; however, 3 of these studies described dissemination for piloting the toolkit and not for full rollout of the toolkit. Common dissemination strategies included sending directly to end users (n=4) [41,46,54,64], social media/blog posting (n=3) [41,45,48], hosting the toolkit online (n=3) [45,48,51], sharing with partner organizations/community representatives (n=3) [45,47,52], continuing education sessions (n=2) [45,53], and presenting at conferences (n=2) [45,52].

Toolkit evaluation methods were described in 12 studies [39,40,42-44,46-48,50,51,54,55], with results described in 11 studies (one study described future evaluation initiatives) [39,40,42-44,47,48,50,51,54,55]. One study evaluated knowledge user engagement but did not evaluate the toolkit itself [45]. Common evaluation methods included surveys (n=7) [40,42,43,47,48,51,54], interviews (n=7) [39,44,46,47,50,54,55], website metrics (n=2) [47,48], observation (n=1) [39], document analysis (n=1) [39], and clinical data (n=1) [44]. No TMFs were explicitly stated as guiding the evaluation procedures. The evaluation outcomes most commonly examined included toolkit usability (n=5) [42,43,49,54,55], acceptability (n=4) [49,51,53,54], use of the toolkit in practice (n=3) [44,48,50], user experience with the toolkit (n=3) [39,46,55], feasibility of toolkit use (n=3) [49,54,56], whether recommendations from knowledge users were adequately incorporated (n=2) [47,48], usefulness of the toolkit (n=2) [40,42], change in knowledge (n=2) [47,50], and increased skills (n=2) [42,50].

### Barriers and Facilitators Identified

Fifteen studies examined barriers and/or facilitators to promoting health behaviors among patients [39-49,51,54,56,57], of which 12 examined these in HCPs [39-41,44,46-49,51,54,56,57]. Barriers corresponding to TDF domains included environmental context and resources (n=9) [42-44,47-49,54,56,57], skills (n=5) [40,41,45,48,57], beliefs about consequences (n=4) [44,48,54,56], social/professional role/identity (n=3) [51,54,56], beliefs about capabilities (n=3) [46,51,56], optimism (n=2) [47,54], knowledge (n=2) [39,57], memory (n=1) [54], and social influences (n=1) [54]. Facilitators corresponding to TDF domains included environmental context and resources (n=4) [40,41,48,57], knowledge (n=4) [41,47,54,57], skills (n=4) [39,41,54,57], beliefs about capabilities (n=2) [41,54], social influences (n=2) [47,48], intentions (n=1) [54], memory (n=1) [39], optimism (n=2) [54,57], and social/professional role/identity (n=2) [44,57]. The most frequently reported barrier was time (n=7) [43,44,47,49,54,56,57], including a lack of time to prescribe and tools that take a long time to use. A description of the domains that were cited in this study along with an example for each domain can be found in Table 2.

**Table 2:** Examples of barriers and facilitators mapped to the Theoretical Domains Framework.

Domain	Definition^a^	Example
Beliefs about capabilities	Acceptance of the truth, reality, or validity about an ability, talent, or facility that a person can put to constructive use	Perceived lack of capability to provide health behavior advice
Beliefs about consequences	Acceptance of the truth, reality, or validity about outcomes of a behavior in a given situation	Belief that prescribing exercise will improve patient health
Environmental context and resources	Any circumstance of a person’s situation or environment that discourages or encourages the development of skills and abilities, independence, social competence, and adaptive behavior	Time to prescribe health behaviors
Intentions	A conscious decision to perform a behavior or a resolve to act in a certain way	Decision to address health behaviors in future consultations with patients
Knowledge	An awareness of the existence of something	Lack of knowledge about the target patient population
Memory, attention, and decision processes	The ability to retain information, focus selectively on aspects of the environment and choose between 2 or more alternatives	Difficulties recalling certain elements when prescribing health behaviors
Optimism	The confidence that things will happen for the best or that desired goals will be attained	Confidence that the practice will lead to beneficial changes in patient behavior
Skills	An ability or proficiency acquired through practice	Need for training on how to prescribe for the patient population
Social influences	Those interpersonal processes that can cause individuals to change their thoughts, feelings, or behaviors	Advocacy by respected health care champions
Social/professional role/identity	A coherent set of behaviors and displayed personal qualities of an individual in a social or work setting	Belief that exercise prescription aligned with their role

^a^Definitions for each domain were extracted verbatim from Atkins et al [38].

**Toolkit Development**

Based on our findings and the recommendations/lessons learned reported by authors of included studies, we have developed a series of recommendations for the development process of toolkits to support HCPs in health behavior change that future toolkit developers may consider (Table 3). This provides a novel contribution, as a previous review noted that there is a lack of information regarding the development of toolkits to change health behaviors in people with chronic conditions [22]. First, in line with how we have operationalized toolkits, we recommend that knowledge users should be engaged from start to finish, as several authors noted that having knowledge user feedback and support was critical for the design and rollout of the toolkit. Next, we recommend that toolkit development should be underpinned by theory, as this can provide guidance for implementers on how to change behaviors. Third, we recommend that while there should be systematic planning for toolkit development, there should be flexibility for changes and iteration as needed, including incorporating feedback into future versions of the toolkit for sustainability of toolkit use. Fourth, we note that developers may consider using virtual communication tools during the toolkit development process. Using virtual communication tools was noted by authors as effective for allowing team members from a wide range of areas to be engaged in toolkit development quickly and without having to travel for meetings. Finally, we suggest that implementers may consider providing additional training opportunities for HCPs to support toolkit use. This may include information on how to use the toolkit and/or information on how to promote health behavior change in the patient population.

**Table 3:** Recommendations for the development process for future toolkits to support health care providers in health behavior change for patients with chronic conditions.

Recommendations	Rationale	Supporting evidence
Knowledge users should be engaged from start to finish	Authors noted that engaging a range of knowledge users was a strength and made the toolkit more applicable to their audience	[39,43-51,54,55]
Toolkit development should be underpinned by behavior change theory	Having a behavior change theory underpinning toolkit development can help guide developers in the design and development of the toolkit	[43,54,55]
Toolkits should be developed using systematic planning with room for flexibility and iterative processes	Allowing space for iterative processes allows for feedback to be incorporated to develop a toolkit best suited to end users	[39,48,52,55,56]
Using virtual communication tools (eg, Zoom) may be beneficial for conducting toolkit development	Using virtual communication methods could overcome barriers like transportation time and geography to engage team members from a range of areas	[45,49,52]
Additional training should be provided to health care providers to support toolkit use	Supplementing the toolkit with additional training (eg, workshops) could help end users feel more knowledgeable and confident using the toolkit	[41,50,54]

### Toolkit Definition

Based on our findings, we propose minimum components required for an intervention to be considered a toolkit. Once these components are met, we recognize that toolkits are flexible in what they can include. Developers should consider the needs of their end users and aims of the toolkit when designing the toolkit. Toolkits at a minimum should:

1. Include materials aimed at improving end user’ ability to conduct the intended behavior (eg, increased health behavior prescription). These may include a variety of materials including educational materials, forms to assist in conducting intended behaviors, etc.
2. Include more than one resource. Toolkits may include different modes of resources (eg, videos and written materials) or they may include one type of resource (eg, written sections), so long as there are multiple resources (eg, for a toolkit containing only written sections, multiple written sections should be included).
3. Be designed with end users’ involvement and needs in mind. Resources should be intentionally designed to address the recommendations and barriers/facilitators of end users.

## Discussion

### Summary of Evidence

This scoping review is the first to examine the TMFs, approaches, and methods used in the development of toolkits to support HCPs in changing the health behaviors of their patients with chronic conditions. This review adds to the literature regarding development of toolkits by presenting information on the “toolkit” concept and offering practical recommendations that can be used by future toolkit developers. We included 19 articles in our review that described toolkit development processes. Toolkits were used for a range of HCP audiences, patient populations, and health behaviors. Included studies on toolkit development were from a range of countries. The number of studies increased over the 18-year publication period, with approximately half published since 2020. While the exact reasoning for this growth in toolkit popularity is unknown, it notably coincides with knowledge mobilization plans being increasingly required in grant applications [65,66].

Reported toolkit development phases typically involved initial exploratory work, iterative toolkit development, dissemination, and evaluation. Exploratory work typically included using literature reviews and/or discussions with knowledge users to identify the need for a toolkit and recommendations for toolkit content to address their needs. These recommendations were collected through surveys, interviews/focus groups, and workshops/design sessions. A previous review similarly noted that these are common methods for informing the development of interventions for HCPs [20]. All studies included knowledge users in the process, with a majority reporting the use of participatory methods. Knowledge users were identified from a range of areas and often leveraged current professional networks and wider networks to recruit a range of knowledge users. This finding is similar to a previous review on the methods for designing interventions to change health care professionals’ behaviors, which found that a majority of studies included user engagement when designing the intervention [20]. While several studies reported using participatory methods to engage knowledge users, only one study reported using a TMF to guide knowledge user engagement. Future studies should consider the use of TMFs to guide engagement of knowledge users in toolkit development to ensure equitable, effective, and acceptable engagement. Additionally, it was unclear in several studies how knowledge users were involved in the participatory component of development, especially regarding the role of knowledge users in study procedures. Future studies using co-approaches should systematically delineate the specific roles assumed by knowledge users across all phases of the research cycle, indicating whether their involvement was confined to discrete tasks (eg, toolkit design) or integrated throughout the project, thereby enhancing transparency and evidencing the authenticity of the co-approach. Only one study [57] provided a citation for the term “toolkit”. Toolkits were often multimodal and toolkit components (eg, videos and forms) varied greatly between studies. Thus, there is a need for a clearer definition and conceptualization of a toolkit. This is especially important in implementation research, as the use of consistent terminology and a clear, shared conceptualization of the intervention being examined (ie, a toolkit) is fundamental to studying, comparing, and improving such interventions [67].

In addition to presenting recommendations for toolkit design and development, our review presented a definition of a toolkit. By defining the minimum components of a toolkit and noting recommendations for how toolkits should be developed, future developers can better consider why they are choosing a toolkit as an intervention strategy and how they are developing the toolkit. As there was a previous lack of description and formal recommendations on methods for developing toolkits in this area [22], this can help future developers guide how toolkits should be developed to best suit the needs of their end users and ensure that they use toolkits in a sustainable and effective way. Future research should consider the design of the toolkit in addition to its development, as multiple studies described involving knowledge users to get feedback on the barriers and facilitators to toolkit use or prescribing health behaviors but largely did not describe theories guiding toolkit design (eg, applying learning theory to toolkit design to ensure end users can best learn from toolkit use). In summary, toolkits should be both designed and developed in a systematic way that is informed by TMFs.

### Limitations

This review was not without limitations. First, articles were limited to those written in English, so toolkits that were developed and reported on in largely non-English speaking countries may have been missed. Further, this review excluded gray literature, which may have resulted in exclusion of literature related to the development of toolkits in nonacademic settings. Finally, the generalizability of findings for health behavior change must be considered. No included toolkits were aimed at reducing smoking, alcohol use, or recreational drug use. Additionally, obesity and neurological conditions made up most of the chronic conditions examined in this review. Thus, as toolkits included generally clustered around exercise behaviors and obesity, the generalizability of our findings to populations and toolkits to other populations and health behaviors may be limited. Further, future research may consider a broader definition of health behaviors, including those related to seeking health services, such as those in the taxonomy of Nudelemen and Shiloh [11]. Future research should examine the methods used in the development of toolkits for these areas.

### Conclusions

Our review demonstrates that toolkits used to support HCPs in health behavior change for patients with chronic conditions vary considerably in their components and audiences. Inclusion of knowledge users was found to be an effective component of toolkit development. Based on our findings, we developed a series of recommendations that future teams can consider for the design and the development process of toolkits. Future research should report on the development and design of toolkits for HCPs that are aimed at reducing smoking, excessive alcohol use, and recreational drug use in patients with chronic conditions. This review has important implications for knowledge translation initiatives, as it defines a “toolkit” to allow for better comparisons and contextualization of toolkits in the future. Additionally, the development of recommendations for both design and development of toolkits allows for toolkits that are more likely to be effectively used and close the knowledge-to-practice gap.

## Appendix

Multimedia Appendix 1 PRISMA-ScR checklist.

Multimedia Appendix 2 Search strategies.

Multimedia Appendix 3 Data extraction template.

Multimedia Appendix 4 Main findings for each included study.

## Abbreviations

COM-B: capability, opportunity, and motivation–behavior
HCP: health care provider
JBI: Joanna Briggs Institute
PCC: population, concept, and context
PRISMA-S: Preferred Reporting Items for Systematic Reviews and Meta-Analyses Search Reporting Extension
PRISMA-ScR: Preferred Reporting Items for Systematic Reviews and Meta-Analyses Extension for Scoping Reviews
TDF: Theoretical Domains Framework
TMF: theories, models, and framework
