# Supplementary material for: Toolkits to Support Health Care Providers in Health Behavior Change for Patients With Chronic Conditions: Scoping Review
Source: JMIR Med Educ. 2026 Oct 26;12:e88653. doi: 10.2196/88653 (PMC13749022; doi:10.2196/88653)
Supplement: Supplementary file 2 [file mededu_v12i1e88653_app3.pdf]

Appendix 3: Data extraction instrument

The data extraction instrument has been adapted from the JBI template source of evidence details, characteristics, and results extraction instrument.

**Scoping Review Details**	
Title:	
Study objective/s:	
Study question/s:	
**Inclusion/Exclusion Criteria**	
Population	
Concept	
Context	
Types of evidence source	
**Evidence source Details and Characteristics**	
Citation details (e.g., authors/date, title, journal, volume, issue, pages)	
Country	
Context	
**Details/Results extracted from source of evidence** (in relation to the concept of the scoping review)	
Toolkit name	
Toolkit aims/purpose	
Healthcare provider audience (e.g., primary care physicians, nurses, etc.)	
Patient population targeted	
Health behaviour (e.g., smoking, physical activity)	
Toolkit mode of delivery (e.g., web-based, pamphlet, etc.)	
Stages of toolkit development and implementation	

Co-production/knowledge user engagement	
Stakeholders/knowledge users engaged (e.g., HCPs, patient partners, subject experts)	
Role of stakeholders (e.g., intervention design, material development, content creation)	
Method of co-production/stakeholder involvement (e.g., focus groups, stakeholder panel)	
Method of stakeholder/knowledge user identification	
Recommendations from stakeholders and how they were addressed	
Learning theory and/or behaviour change theory	
Theories, Models, and/or Frameworks applied	
How theories, models, and/or frameworks were applied	
Toolkit componentry	
Tailoring for population (e.g., age considerations, cultural adaptations, clinical needs (e.g., for patients with more severe disability))	
Facilitators	
Barriers	
Methods for cascading toolkit	
Methods of measurement/evaluation (e.g., knowledge increase, use, behaviour change)	
Outcomes (e.g., effectiveness)	
Recommendations provided/lessons learned	
