# Supplementary material for: Toolkits to Support Health Care Providers in Health Behavior Change for Patients With Chronic Conditions: Scoping Review
Source: JMIR Med Educ. 2026 Oct 26;12:e88653. doi: 10.2196/88653 (PMC13749022; doi:10.2196/88653)
Supplement: Supplementary file 3 [file mededu_v12i1e88653_app2.docx]

**Appendix II – Search Strategy**

**Embase Classic - Embase <1947 to 2026 July 30>**

| 1 | tool*.ti. |
| --- | --- |
| 2 | "Exercise and Depression Toolkit".ti,ab. |
| 3 | "5As obesity tools".ti,ab. |
| 4 | quality assessment tool/ or "tool use"/ |
| 5 | 1 or 2 or 3 or 4 |
| 6 | translational research/ or motivational interviewing/ or transtheoretical model/ |
| 7 | "Theoretical Domains framework".ti,ab,kw. |
| 8 | Social Learning Theory/ or "Theory of Planned Behavior"/ |
| 9 | Agree II instrument.ti,ab,kw. |
| 10 | ((knowledge or research) adj2 translat*).ti,ab,kw. |
| 11 | "Knowledge To Action framework".ti,ab,kw. |
| 12 | "Theory of Reasoned Action"/ or Social Cognitive Theory/ or co-design*.tw,kw. |
| 13 | ("social cognitive theory" or "transtheoretical model" or "self determination theory" or "motivational interviewing" or "theory of planned behavio?r" or "social learning theory" or "theory of reasoned action" or "stages of change" or "health belief model" or "behavio?r change wheel" or "personalization theory" or "social practice theory" or nudg* or "stages of change").tw,kw. |
| 14 | or/6-13 |
| 15 | health education/ or behavior change/ or behavior modification/ or health promotion/ or healthy lifestyle/ or exp health behavior/ |
| 16 | ((health or lifestyle or behavio?r*) adj2 (interv* or chang* or modif* or prevent* or promot* or model* or framework* or method* or approach*)).tw,kw. |
| 17 | 15 or 16 |
| 18 | exercise/ or physical activity/ or sleep/ or smoking/ or alcoholism/ or heavy drinking/ or nutrition/ or diet/ or body weight management/ or obesity/ or drinking behavior/ |
| 19 | (weight or obese or obesity or obeseness or over-weight or sleep* or sedentary or exercis* or "physical activ*" or smoking or nutrition or alcohol* or "heavy drinking" or diet* or lifestyle).tw,kw. |
| 20 | 18 or 19 |
| 21 | (("knowledge translation" or interven* or framework* or method* or approach*) adj1 (health* adj2 (behavio?r or chang* or modif* or promot* or lifestyle or prevent*))).tw. |
| 22 | health care personnel/ |
| 23 | ((health-care or hospital or medical or allied-health) adj1 (practitioner* or professional* or provider* or personnel or support worker*)).tw,kw. |
| 24 | health practitioner/ |
| 25 | paramedical personnel/ |
| 26 | (para-medical adj1 (personnel or assistant* or professional* or manpower)).tw,kw. |
| 27 | 22 or 23 or 24 or 25 or 26 |
| 28 | 5 and 14 and 17 and 20 and 27 |
| 29 | 5 and 14 and 17 and 20 |
| 30 | 5 and 17 and 20 |
| 31 | 5 and 17 and 20 and 27 |
| 32 | 5 and 14 and 20 |
| 33 | 14 and 17 and 20 and 27 |
| 34 | 5 and 20 and 21 |
| 35 | 5 and 20 and 21 and 27 |
| 36 | 5 and 21 and 27 |
| 37 | 5 and 21 |
| 38 | 5 and 14 and 17 |
| 39 | or/28-38 |
| 40 | Animals/ not (Animals/ and Humans/) |
| 41 | 39 not 40 |
| 42 | remove duplicates from 41 |

| **APA PsycInfo <1806 to July 30 2026>** | |
| --- | --- |
| 1 | tool*.ti. |
| 2 | intervention/ |
| 3 | 1 or 2 |
| 4 | applied behavior analysis/ or behavior analysis/ or behavior change/ or behavior problems/ or learning theory/ |
| 5 | motivational interviewing/ |
| 6 | transtheoretical model/ or "stages of change"/ |
| 7 | "Theoretical Domains framework".ti,ab. |
| 8 | Learning Theory/ and Social Learning/ |
| 9 | reasoned action/ |
| 10 | Agree II instrument.ti,ab. |
| 11 | ((knowledge or research) adj2 translat*).ti,ab. |
| 12 | "Knowledge To Action framework".ti,ab. |
| 13 | reasoned action/ |
| 14 | Social Cognition/ |
| 15 | co-design*.ti,ab. |
| 16 | ("social cognitive theory" or "transtheoretical model" or "self determination theory" or "motivational interviewing" or "theory of planned behavio?r" or "social learning theory" or "theory of reasoned action" or "stages of change" or "health belief model" or "behavio?r change wheel" or "personalization theory" or "social practice theory" or nudg* or "stages of change").ti,ab. |
| 17 | or/4-16 |
| 18 | health education/ |
| 19 | behavior change/ or readiness to change/ or "stages of change"/ or behavior modification/ or lifestyle changes/ |
| 20 | behavior modification/ or behavior therapy/ |
| 21 | health promotion/ or client education/ or health attitudes/ or health awareness/ or health behavior/ or health knowledge/ |
| 22 | health behavior/ or health risk behavior/ or healthy eating/ or preventive health behavior/ |
| 23 | ((health or lifestyle or behavio?r*) adj2 (interv* or chang* or modif* or prevent* or promot* or model* or framework* or method* or approach*)).ti,ab. |
| 24 | or/18-23 |
| 25 | exercise/ or aerobic exercise/ or weightlifting/ or yoga/ |
| 26 | physical activity/ or active living/ |
| 27 | sleep/ or sleep quality/ |
| 28 | Smoking Cessation/ or Tobacco Smoking/ or Smoking Prevention/ |
| 29 | alcoholism/ or "alcohol use"/ or "alcohol use attitudes"/ or nutritional deficiencies/ |
| 30 | "Alcohol Use"/ or Drinking Behavior/ or Binge Drinking/ |
| 31 | nutrition/ or diets/ or food/ or healthy eating/ |
| 32 | Obesity/ or Overweight/ or Weight Control/ or Body Weight/ |
| 33 | (weight or obese or obesity or obeseness or over-weight or sleep* or sedentary or exercis* or "physical activ*" or smoking or nutrition or alcohol* or "heavy drinking" or diet* or lifestyle).ti,ab. |
| 34 | or/25-33 |
| 35 | (("knowledge translation" or interven* or framework* or method* or approach*) adj1 (health* adj2 (behavio?r or chang* or modif* or promot* or lifestyle or prevent*))).ti,ab. |
| 36 | health personnel/ or allied health personnel/ or medical personnel/ |
| 37 | ((health-care or hospital or medical or allied-health) adj1 (practitioner* or professional* or provider* or personnel or support worker*)).ti,ab. |
| 38 | 36 or 37 |
| 39 | 3 and 17 and 24 and 34 and 38 |
| 40 | 3 and 17 and 24 and 34 |
| 41 | 3 and 24 and 34 |
| 42 | 3 and 24 and 34 and 38 |
| 43 | 3 and 17 and 34 |
| 44 | 17 and 24 and 34 and 38 |
| 45 | 3 and 34 and 35 |
| 46 | 3 and 34 and 35 and 38 |
| 47 | 3 and 35 and 38 |
| 48 | 3 and 35 |
| 49 | 3 and 17 and 24 |
| 50 | or/39-49 |
| 51 | 39 or 40 or 42 or 43 or 44 or 45 or 46 or 47 or 48 or 49 |
| 52 | (Developing tools to support patients and healthcare providers when in conversation about obesity).m_titl. |
| 53 | Animals/ not (Animals/ and Humans/) |
| 54 | 51 not 53 |
| 55 | remove duplicates from 54 |

| **Ovid MEDLINE(R) ALL <1946 to July 30 2026>** | |
| --- | --- |
|  |  |
| 1 | tool*.ti. |
| 2 | exp "Tool Use Behavior"/ |
| 3 | 1 or 2 |
| 4 | Translational Research, Biomedical/ |
| 5 | Motivational Interviewing/ |
| 6 | transtheoretical model/ |
| 7 | "Theoretical Domains framework".tw,kf. |
| 8 | Conditioning, Psychological/ |
| 9 | "theory of planned behavior"/ |
| 10 | Agree II instrument.tw,kf. |
| 11 | ((knowledge or research) adj2 translat*).tw,kf. |
| 12 | "Knowledge To Action framework".tw,kf. |
| 13 | Psychological Theory/ |
| 14 | co-design*.tw,kf. |
| 15 | ("social cognitive theory" or "transtheoretical model" or "self determination theory" or "motivational interviewing" or "theory of planned behavio?r" or "social learning theory" or "theory of reasoned action" or "stages of change" or "health belief model" or "behavio?r change wheel" or "personalization theory" or "social practice theory" or nudg* or "stages of change").tw,kf. |
| 16 | or/4-15 |
| 17 | health education/ or consumer health information/ or health promotion/ or patient education as topic/ or smoking prevention/ |
| 18 | health behavior/ or health risk behaviors/ or "treatment adherence and compliance"/ |
| 19 | Behavior Therapy/ |
| 20 | health promotion/ or healthy people programs/ |
| 21 | healthy lifestyle/ |
| 22 | ((health or lifestyle or behavio?r*) adj2 (interv* or chang* or modif* or prevent* or promot* or model* or framework* or method* or approach*)).tw,kf. |
| 23 | or/17-22 |
| 24 | Exercise/ |
| 25 | Sleep/ |
| 26 | smoking/ or smoking reduction/ or smoking prevention/ or sleep hygiene/ or smoking cessation/ or smoking reduction/ or "tobacco use cessation"/ |
| 27 | weight reduction programs/ or diet, healthy/ |
| 28 | Alcoholism/ |
| 29 | alcohol drinking/ or binge drinking/ |
| 30 | nutrition assessment/ |
| 31 | Diet/ |
| 32 | obesity/ or overweight/ |
| 33 | (weight or obese or obesity or obeseness or over-weight or sleep* or sedentary or exercis* or "physical activ*" or smoking or nutrition or alcohol* or "heavy drinking" or diet* or lifestyle).tw,kf. |
| 34 | or/24-33 |
| 35 | (("knowledge translation" or interven* or framework* or method* or approach*) adj1 (health* adj2 (behavio?r or chang* or modif* or promot* or lifestyle or prevent*))).tw. |
| 36 | Health Personnel/ |
| 37 | ((health-care or hospital or medical or allied-health) adj1 (practitioner* or professional* or provider* or personnel or support worker*)).tw,kf. |
| 38 | 36 or 37 |
| 39 | 3 and 16 and 23 and 34 and 38 |
| 40 | 3 and 16 and 23 and 34 |
| 41 | 3 and 23 and 34 |
| 42 | 3 and 23 and 34 and 38 |
| 43 | 3 and 16 and 34 |
| 44 | 16 and 23 and 34 and 38 |
| 45 | 3 and 34 and 35 |
| 46 | 3 and 34 and 35 and 38 |
| 47 | 3 and 35 and 38 |
| 48 | 3 and 35 |
| 49 | 3 and 16 and 23 |
| 50 | or/39-49 |
| 51 | Animals/ not (Animals/ and Humans/) |
| 52 | 50 not 51 |
| 53 | remove duplicates from 52 |
| 54 | A collaborative process for developing a weight management toolkit for general practitioners.m_titl. |
| 55 | 53 or 54 |

**Scopus - July 30, 2026**

( ( ( TITLE-ABS-KEY ( tool* OR "5As obesity tools" ) ) AND ( TITLE-ABS-KEY ( ( ( health OR lifestyle OR behavio*r ) W/2 ( interv* OR chang* OR modif* OR prevent* OR promot* OR model* OR framework* OR method* OR approach OR educat* ) ) ) ) AND ( TITLE-ABS-KEY ( ( "social cognitive theory" OR "transtheoretical model" OR "self determination theory" OR "motivational interviewing" OR "theory of planned behavio*r" OR "social learning theory" OR "theory of reasoned action" OR "stages of change" OR "health belief model" OR "behavio*r change wheel" OR "personalization theory" OR "social practice theory" OR nudge OR "stages of change" OR "translational research" OR "Theoretical Domains framework" OR "Agree II instrument" ) ) ) ) OR ( ( TITLE-ABS-KEY ( tool* OR "5As obesity tools" ) ) AND ( TITLE-ABS-KEY ( ( "social cognitive theory" OR "transtheoretical model" OR "self determination theory" OR "motivational interviewing" OR "theory of planned behavio*r" OR "social learning theory" OR "theory of reasoned action" OR "stages of change" OR "health belief model" OR "behavio*r change wheel" OR "personalization theory" OR "social practice theory" OR nudge OR "stages of change" OR "translational research" OR "Theoretical Domains framework" OR "Agree II instrument" ) ) ) AND ( TITLE-ABS-KEY ( ( ( health OR lifestyle OR behavio*r ) W/2 ( interv* OR chang* OR modif* OR prevent* OR promot* OR model* OR framework* OR method* OR approach OR educat* ) ) ) ) AND ( TITLE-ABS-KEY ( ( weight OR obese OR obesity OR obeseness OR over-weight OR sleep* OR sedentary OR exercis* OR "physical activ*" OR smoking OR nutrition OR alcohol* OR "heavy drinking" OR diet* OR lifestyle ) ) ) AND ( TITLE-ABS-KEY ( ( healthcare W/2 provider* ) ) ) ) OR ( ( TITLE-ABS-KEY ( ( healthcare W/2 provider* ) ) ) AND ( TITLE-ABS-KEY ( ( weight OR obese OR obesity OR obeseness OR over-weight OR sleep* OR sedentary OR exercis* OR "physical activ*" OR smoking OR nutrition OR alcohol* OR "heavy drinking" OR diet* OR lifestyle ) ) ) AND ( TITLE-ABS-KEY ( tool* OR "5As obesity tools" ) ) ) ) OR ( TITLE ( developing AND tools AND to AND support AND patients AND healthcare AND providers AND when AND in AND conversation AND about AND obesity ) )

**Web of Science – July 30, 2026**

\# Searches:

1: TI=(tool)

2: AB=(tool)

3: #2 OR #1

4: TI=(("social cognitive theory" or "transtheoretical model" or "self determination theory" or "motivational interviewing" or "theory of planned behavio$r" or "social learning theory" or "theory of reasoned action" or "stages of change" or "health belief model" or "behavio$r change wheel" or "personalization theory" or "social practice theory" or nudge or "stages of change" or “translational research” or “Theoretical Domains framework” or “Agree II instrument” ).)

5: AB=(("social cognitive theory" or "transtheoretical model" or "self determination theory" or "motivational interviewing" or "theory of planned behavio$r" or "social learning theory" or "theory of reasoned action" or "stages of change" or "health belief model" or "behavio$r change wheel" or "personalization theory" or "social practice theory" or nudge or "stages of change" or “translational research” or “Theoretical Domains framework” or “Agree II instrument” ).)

6: #5 OR #4

7: TI=((health or lifestyle or behavio$r*) NEAR/2 (interv* or chang* or modif* or prevent* or promot* or model* or framework* or method* or approach or educat*))

8: AB=((health or lifestyle or behavio$r*) NEAR/2 (interv* or chang* or modif* or prevent* or promot* or model* or framework* or method* or approach or educat*))

9: #7 OR #8

10: TI=(weight or obese or obesity or obeseness or over-weight or sleep* or sedentary or exercise or "physical activity" or smoking or nutrition or alcohol* or "heavy drinking" or diet* or lifestyle)

11: AB=(weight or obese or obesity or obeseness or over-weight or sleep* or sedentary or exercise or "physical activity" or smoking or nutrition or alcohol* or "heavy drinking" or diet* or lifestyle)

12: #10 OR #11

13: #12 AND #9 AND #6 AND #3

14: tI=("health practitioner" OR "allied health" or "patient education")

15: AB=("health practitioner" OR "allied health" or "patient education")

16: #15 OR #14

17: #16 AND #12 AND #9 AND #6 AND #3

18: #16 AND #3 AND #9

19: #3 AND #12 AND #16

20: #19 OR #18 OR #17 OR #13

**CINAHL – August 24, 2026**

**Search:** ((EM 202411-)) AND ((((((TI tool* OR AB tool*)) OR (((MH "Quality Assessment")))) AND (((TI ( ("social cognitive theory" or"transtheoretical model" or "self determination theory" or"motivational interviewing" or"theory of planned behavio#r" or"social learning theory" or "theory of reasoned action" or "stages of change" or "health belief model" or"behavio#r change wheel" or"personalization theory" or "social practice theory" or nudg* or "stages of change"). ) OR AB ( ("social cognitive theory" or"transtheoretical model" or "self determination theory" or"motivational interviewing" or"theory of planned behavio#r" or"social learning theory" or "theory of reasoned action" or "stages of change" or "health belief model" or"behavio#r change wheel" or"personalization theory" or "social practice theory" or nudg* or "stages of change"). ))) OR ((TI co-design* OR AB co-design*)) OR (((MH "Bandura's Social Cognitive Theory"))) OR (((MH "Ajzen-Fishbein Theory of Reasoned Action"))) OR ((TI "Knowledge To Action framework" OR AB "Knowledge To Action framework")) OR ((TI ( ((knowledge or research) N2translat*) ) OR AB ( ((knowledge or research) N2 translat*) ))) OR ((TI "Agree II instrument*" OR AB"Agree II instrument*")) OR (((MH "Ajzen's Theory of Planned Behavior"))) OR (((MH "Social Learning Theory"))) OR ((TI "Theoretical Domains framework" OR AB "Theoretical Domains framework")) OR (((MH "Transtheoretical Stages of Change Model"))) OR (((MH "Motivational Interviewing"))) OR (((MH "Translational Medical Research")))) AND (((MH "Health Education")) OR ((MH "Behavioral Changes")) OR ((MH "Behavior Modification")) OR ((MH "Health Promotion")) ORS22 OR ((MH "Health Behavior")) OR (TI ( ((health or lifestyle or behavio?r*) N2 (interv* or chang* or modif* or prevent* or promot* or model* or framework* or method* or approach*)) ) OR AB ( ((health or lifestyle or behavio?r*) N2 (interv*or chang* or modif* or prevent* or promot* or model* or framework* or method* or approach*)) )))) OR ((((TI tool* OR AB tool*)) OR (((MH "Quality Assessment")))) AND (TI ( (("knowledge translation" orinterven* or framework* or method*or approach*) N1 (health* N2 (behavio?r or chang* or modif* orpromot* or lifestyle or prevent*))). )OR AB ( (("knowledge translation"or interven* or framework* or method* or approach*) N1 (health*N2 (behavio?r or chang* or modif*or promot* or lifestyle or prevent*))).))) OR ((((TI tool* OR AB tool*)) OR (((MH "Quality Assessment")))) AND (((TI ( ("social cognitive theory" or"transtheoretical model" or "self determination theory" or"motivational interviewing" or"theory of planned behavio#r" or"social learning theory" or "theory of reasoned action" or "stages of change" or "health belief model" or"behavio#r change wheel" or"personalization theory" or "social practice theory" or nudg* or "stages of change"). ) OR AB ( ("social cognitive theory" or"transtheoretical model" or "self determination theory" or"motivational interviewing" or"theory of planned behavio#r" or"social learning theory" or "theory of reasoned action" or "stages of change" or "health belief model" or"behavio#r change wheel" or"personalization theory" or "social practice theory" or nudg* or "stages of change"). ))) OR ((TI co-design* OR AB co-design*)) OR (((MH "Bandura's Social Cognitive Theory"))) OR (((MH "Ajzen-Fishbein Theory of Reasoned Action"))) OR ((TI "Knowledge To Action framework" OR AB "Knowledge To Action framework")) OR ((TI ( ((knowledge or research) N2translat*) ) OR AB ( ((knowledge or research) N2 translat*) ))) OR ((TI "Agree II instrument*" OR AB"Agree II instrument*")) OR (((MH "Ajzen's Theory of Planned Behavior"))) OR (((MH "Social Learning Theory"))) OR ((TI "Theoretical Domains framework" OR AB "Theoretical Domains framework")) OR (((MH "Transtheoretical Stages of Change Model"))) OR (((MH "Motivational Interviewing"))) OR (((MH "Translational Medical Research")))) AND (((MH "Health Education")) OR ((MH "Behavioral Changes")) OR ((MH "Behavior Modification")) OR ((MH "Health Promotion")) ORS22 OR ((MH "Health Behavior")) OR (TI ( ((health or lifestyle or behavio?r*) N2 (interv* or chang* or modif* or prevent* or promot* or model* or framework* or method* or approach*)) ) OR AB ( ((health or lifestyle or behavio?r*) N2 (interv*or chang* or modif* or prevent* or promot* or model* or framework* or method* or approach*)) ))) AND (((MH "Exercise")) OR ((MH "Physical Activity")) OR ((MH "Sleep")) OR ((MH "Smoking")) ORS30 OR ((MH "Drinking Behavior")) OR ((MH "Nutrition")) OR ((MH "Diet")) ORS34 OR ((MH "Obesity")) OR (TI ( (weight or obese or obesity or obeseness or over-weight or sleep*or sedentary or exercis* or"physical activ*" or smoking or nutrition or alcohol* or "heavy drinking" or diet* or lifestyle). ) ORAB ( (weight or obese or obesity or obeseness or over-weight or sleep*or sedentary or exercis* or"physical activ*" or smoking or nutrition or alcohol* or "heavy drinking" or diet* or lifestyle). ))) AND (((MH "Health Personnel")) OR (TI ( ((health-care or hospital or medical or allied-health) N1(practitioner* or professional* or provider* or personnel or support worker*)) ) OR AB ( ((health-care or hospital or medical or allied-health)N1 (practitioner* or professional* or provider* or personnel or support worker*)) )))) OR ((((TI tool* OR AB tool*)) OR (((MH "Quality Assessment")))) AND (((TI ( ("social cognitive theory" or"transtheoretical model" or "self determination theory" or"motivational interviewing" or"theory of planned behavio#r" or"social learning theory" or "theory of reasoned action" or "stages of change" or "health belief model" or"behavio#r change wheel" or"personalization theory" or "social practice theory" or nudg* or "stages of change"). ) OR AB ( ("social cognitive theory" or"transtheoretical model" or "self determination theory" or"motivational interviewing" or"theory of planned behavio#r" or"social learning theory" or "theory of reasoned action" or "stages of change" or "health belief model" or"behavio#r change wheel" or"personalization theory" or "social practice theory" or nudg* or "stages of change"). ))) OR ((TI co-design* OR AB co-design*)) OR (((MH "Bandura's Social Cognitive Theory"))) OR (((MH "Ajzen-Fishbein Theory of Reasoned Action"))) OR ((TI "Knowledge To Action framework" OR AB "Knowledge To Action framework")) OR ((TI ( ((knowledge or research) N2translat*) ) OR AB ( ((knowledge or research) N2 translat*) ))) OR ((TI "Agree II instrument*" OR AB"Agree II instrument*")) OR (((MH "Ajzen's Theory of Planned Behavior"))) OR (((MH "Social Learning Theory"))) OR ((TI "Theoretical Domains framework" OR AB "Theoretical Domains framework")) OR (((MH "Transtheoretical Stages of Change Model"))) OR (((MH "Motivational Interviewing"))) OR (((MH "Translational Medical Research")))) AND (((MH "Health Education")) OR ((MH "Behavioral Changes")) OR ((MH "Behavior Modification")) OR ((MH "Health Promotion")) ORS22 OR ((MH "Health Behavior")) OR (TI ( ((health or lifestyle or behavio?r*) N2 (interv* or chang* or modif* or prevent* or promot* or model* or framework* or method* or approach*)) ) OR AB ( ((health or lifestyle or behavio?r*) N2 (interv*or chang* or modif* or prevent* or promot* or model* or framework* or method* or approach*)) ))) AND (((MH "Exercise")) OR ((MH "Physical Activity")) OR ((MH "Sleep")) OR ((MH "Smoking")) ORS30 OR ((MH "Drinking Behavior")) OR ((MH "Nutrition")) OR ((MH "Diet")) ORS34 OR ((MH "Obesity")) OR (TI ( (weight or obese or obesity or obeseness or over-weight or sleep*or sedentary or exercis* or"physical activ*" or smoking or nutrition or alcohol* or "heavy drinking" or diet* or lifestyle). ) ORAB ( (weight or obese or obesity or obeseness or over-weight or sleep*or sedentary or exercis* or"physical activ*" or smoking or nutrition or alcohol* or "heavy drinking" or diet* or lifestyle). )))) OR ((((TI tool* OR AB tool*)) OR (((MH "Quality Assessment")))) AND (((MH "Health Education")) OR ((MH "Behavioral Changes")) OR ((MH "Behavior Modification")) OR ((MH "Health Promotion")) ORS22 OR ((MH "Health Behavior")) OR (TI ( ((health or lifestyle or behavio?r*) N2 (interv* or chang* or modif* or prevent* or promot* or model* or framework* or method* or approach*)) ) OR AB ( ((health or lifestyle or behavio?r*) N2 (interv*or chang* or modif* or prevent* or promot* or model* or framework* or method* or approach*)) ))) AND (((MH "Exercise")) OR ((MH "Physical Activity")) OR ((MH "Sleep")) OR ((MH "Smoking")) ORS30 OR ((MH "Drinking Behavior")) OR ((MH "Nutrition")) OR ((MH "Diet")) ORS34 OR ((MH "Obesity")) OR (TI ( (weight or obese or obesity or obeseness or over-weight or sleep*or sedentary or exercis* or"physical activ*" or smoking or nutrition or alcohol* or "heavy drinking" or diet* or lifestyle). ) ORAB ( (weight or obese or obesity or obeseness or over-weight or sleep*or sedentary or exercis* or"physical activ*" or smoking or nutrition or alcohol* or "heavy drinking" or diet* or lifestyle). )))) OR ((((TI tool* OR AB tool*)) OR (((MH "Quality Assessment")))) AND (((MH "Health Education")) OR ((MH "Behavioral Changes")) OR ((MH "Behavior Modification")) OR ((MH "Health Promotion")) ORS22 OR ((MH "Health Behavior")) OR (TI ( ((health or lifestyle or behavio?r*) N2 (interv* or chang* or modif* or prevent* or promot* or model* or framework* or method* or approach*)) ) OR AB ( ((health or lifestyle or behavio?r*) N2 (interv*or chang* or modif* or prevent* or promot* or model* or framework* or method* or approach*)) ))) AND (((MH "Exercise")) OR ((MH "Physical Activity")) OR ((MH "Sleep")) OR ((MH "Smoking")) ORS30 OR ((MH "Drinking Behavior")) OR ((MH "Nutrition")) OR ((MH "Diet")) ORS34 OR ((MH "Obesity")) OR (TI ( (weight or obese or obesity or obeseness or over-weight or sleep*or sedentary or exercis* or"physical activ*" or smoking or nutrition or alcohol* or "heavy drinking" or diet* or lifestyle). ) ORAB ( (weight or obese or obesity or obeseness or over-weight or sleep*or sedentary or exercis* or"physical activ*" or smoking or nutrition or alcohol* or "heavy drinking" or diet* or lifestyle). ))) AND (((MH "Health Personnel")) OR (TI ( ((health-care or hospital or medical or allied-health) N1(practitioner* or professional* or provider* or personnel or support worker*)) ) OR AB ( ((health-care or hospital or medical or allied-health)N1 (practitioner* or professional* or provider* or personnel or support worker*)) )))) OR ((((TI tool* OR AB tool*)) OR (((MH "Quality Assessment")))) AND (((TI ( ("social cognitive theory" or"transtheoretical model" or "self determination theory" or"motivational interviewing" or"theory of planned behavio#r" or"social learning theory" or "theory of reasoned action" or "stages of change" or "health belief model" or"behavio#r change wheel" or"personalization theory" or "social practice theory" or nudg* or "stages of change"). ) OR AB ( ("social cognitive theory" or"transtheoretical model" or "self determination theory" or"motivational interviewing" or"theory of planned behavio#r" or"social learning theory" or "theory of reasoned action" or "stages of change" or "health belief model" or"behavio#r change wheel" or"personalization theory" or "social practice theory" or nudg* or "stages of change"). ))) OR ((TI co-design* OR AB co-design*)) OR (((MH "Bandura's Social Cognitive Theory"))) OR (((MH "Ajzen-Fishbein Theory of Reasoned Action"))) OR ((TI "Knowledge To Action framework" OR AB "Knowledge To Action framework")) OR ((TI ( ((knowledge or research) N2translat*) ) OR AB ( ((knowledge or research) N2 translat*) ))) OR ((TI "Agree II instrument*" OR AB"Agree II instrument*")) OR (((MH "Ajzen's Theory of Planned Behavior"))) OR (((MH "Social Learning Theory"))) OR ((TI "Theoretical Domains framework" OR AB "Theoretical Domains framework")) OR (((MH "Transtheoretical Stages of Change Model"))) OR (((MH "Motivational Interviewing"))) OR (((MH "Translational Medical Research")))) AND (((MH "Exercise")) OR ((MH "Physical Activity")) OR ((MH "Sleep")) OR ((MH "Smoking")) ORS30 OR ((MH "Drinking Behavior")) OR ((MH "Nutrition")) OR ((MH "Diet")) ORS34 OR ((MH "Obesity")) OR (TI ( (weight or obese or obesity or obeseness or over-weight or sleep*or sedentary or exercis* or"physical activ*" or smoking or nutrition or alcohol* or "heavy drinking" or diet* or lifestyle). ) ORAB ( (weight or obese or obesity or obeseness or over-weight or sleep*or sedentary or exercis* or"physical activ*" or smoking or nutrition or alcohol* or "heavy drinking" or diet* or lifestyle). )))) OR ((((TI ( ("social cognitive theory" or"transtheoretical model" or "self determination theory" or"motivational interviewing" or"theory of planned behavio#r" or"social learning theory" or "theory of reasoned action" or "stages of change" or "health belief model" or"behavio#r change wheel" or"personalization theory" or "social practice theory" or nudg* or "stages of change"). ) OR AB ( ("social cognitive theory" or"transtheoretical model" or "self determination theory" or"motivational interviewing" or"theory of planned behavio#r" or"social learning theory" or "theory of reasoned action" or "stages of change" or "health belief model" or"behavio#r change wheel" or"personalization theory" or "social practice theory" or nudg* or "stages of change"). ))) OR ((TI co-design* OR AB co-design*)) OR (((MH "Bandura's Social Cognitive Theory"))) OR (((MH "Ajzen-Fishbein Theory of Reasoned Action"))) OR ((TI "Knowledge To Action framework" OR AB "Knowledge To Action framework")) OR ((TI ( ((knowledge or research) N2translat*) ) OR AB ( ((knowledge or research) N2 translat*) ))) OR ((TI "Agree II instrument*" OR AB"Agree II instrument*")) OR (((MH "Ajzen's Theory of Planned Behavior"))) OR (((MH "Social Learning Theory"))) OR ((TI "Theoretical Domains framework" OR AB "Theoretical Domains framework")) OR (((MH "Transtheoretical Stages of Change Model"))) OR (((MH "Motivational Interviewing"))) OR (((MH "Translational Medical Research")))) AND (((MH "Health Education")) OR ((MH "Behavioral Changes")) OR ((MH "Behavior Modification")) OR ((MH "Health Promotion")) ORS22 OR ((MH "Health Behavior")) OR (TI ( ((health or lifestyle or behavio?r*) N2 (interv* or chang* or modif* or prevent* or promot* or model* or framework* or method* or approach*)) ) OR AB ( ((health or lifestyle or behavio?r*) N2 (interv*or chang* or modif* or prevent* or promot* or model* or framework* or method* or approach*)) ))) AND (((MH "Exercise")) OR ((MH "Physical Activity")) OR ((MH "Sleep")) OR ((MH "Smoking")) ORS30 OR ((MH "Drinking Behavior")) OR ((MH "Nutrition")) OR ((MH "Diet")) ORS34 OR ((MH "Obesity")) OR (TI ( (weight or obese or obesity or obeseness or over-weight or sleep*or sedentary or exercis* or"physical activ*" or smoking or nutrition or alcohol* or "heavy drinking" or diet* or lifestyle). ) ORAB ( (weight or obese or obesity or obeseness or over-weight or sleep*or sedentary or exercis* or"physical activ*" or smoking or nutrition or alcohol* or "heavy drinking" or diet* or lifestyle). ))) AND (((MH "Health Personnel")) OR (TI ( ((health-care or hospital or medical or allied-health) N1(practitioner* or professional* or provider* or personnel or support worker*)) ) OR AB ( ((health-care or hospital or medical or allied-health)N1 (practitioner* or professional* or provider* or personnel or support worker*)) )))) OR ((((TI tool* OR AB tool*)) OR (((MH "Quality Assessment")))) AND (((MH "Exercise")) OR ((MH "Physical Activity")) OR ((MH "Sleep")) OR ((MH "Smoking")) ORS30 OR ((MH "Drinking Behavior")) OR ((MH "Nutrition")) OR ((MH "Diet")) ORS34 OR ((MH "Obesity")) OR (TI ( (weight or obese or obesity or obeseness or over-weight or sleep*or sedentary or exercis* or"physical activ*" or smoking or nutrition or alcohol* or "heavy drinking" or diet* or lifestyle). ) ORAB ( (weight or obese or obesity or obeseness or over-weight or sleep*or sedentary or exercis* or"physical activ*" or smoking or nutrition or alcohol* or "heavy drinking" or diet* or lifestyle). ))) AND (TI ( (("knowledge translation" orinterven* or framework* or method*or approach*) N1 (health* N2 (behavio?r or chang* or modif* orpromot* or lifestyle or prevent*))). )OR AB ( (("knowledge translation"or interven* or framework* or method* or approach*) N1 (health*N2 (behavio?r or chang* or modif*or promot* or lifestyle or prevent*))).))) OR ((((TI tool* OR AB tool*)) OR (((MH "Quality Assessment")))) AND (((MH "Exercise")) OR ((MH "Physical Activity")) OR ((MH "Sleep")) OR ((MH "Smoking")) ORS30 OR ((MH "Drinking Behavior")) OR ((MH "Nutrition")) OR ((MH "Diet")) ORS34 OR ((MH "Obesity")) OR (TI ( (weight or obese or obesity or obeseness or over-weight or sleep*or sedentary or exercis* or"physical activ*" or smoking or nutrition or alcohol* or "heavy drinking" or diet* or lifestyle). ) ORAB ( (weight or obese or obesity or obeseness or over-weight or sleep*or sedentary or exercis* or"physical activ*" or smoking or nutrition or alcohol* or "heavy drinking" or diet* or lifestyle). ))) AND (TI ( (("knowledge translation" orinterven* or framework* or method*or approach*) N1 (health* N2 (behavio?r or chang* or modif* orpromot* or lifestyle or prevent*))). )OR AB ( (("knowledge translation"or interven* or framework* or method* or approach*) N1 (health*N2 (behavio?r or chang* or modif*or promot* or lifestyle or prevent*))).)) AND (((MH "Health Personnel")) OR (TI ( ((health-care or hospital or medical or allied-health) N1(practitioner* or professional* or provider* or personnel or support worker*)) ) OR AB ( ((health-care or hospital or medical or allied-health)N1 (practitioner* or professional* or provider* or personnel or support worker*)) )))) OR ((((TI tool* OR AB tool*)) OR (((MH "Quality Assessment")))) AND (TI ( (("knowledge translation" orinterven* or framework* or method*or approach*) N1 (health* N2 (behavio?r or chang* or modif* orpromot* or lifestyle or prevent*))). )OR AB ( (("knowledge translation"or interven* or framework* or method* or approach*) N1 (health*N2 (behavio?r or chang* or modif*or promot* or lifestyle or prevent*))).)) AND (((MH "Health Personnel")) OR (TI ( ((health-care or hospital or medical or allied-health) N1(practitioner* or professional* or provider* or personnel or support worker*)) ) OR AB ( ((health-care or hospital or medical or allied-health)N1 (practitioner* or professional* or provider* or personnel or support worker*)) ))))))
