# Supplementary material for: Toolkits to Support Health Care Providers in Health Behavior Change for Patients With Chronic Conditions: Scoping Review
Source: JMIR Med Educ. 2026 Oct 26;12:e88653. doi: 10.2196/88653 (PMC13749022; doi:10.2196/88653)
Supplement: Supplementary file 4 [file mededu_v12i1e88653_app4.docx]

Appendix 4. Main findings for each included study

| Study | Theories, Models & Frameworks (TMFs) Used | Operationalization of TMFs | Development Phases | Barriers | Facilitators |
| --- | --- | --- | --- | --- | --- |
| Arbour-Nicitopoulos 2013 | NR | N/A | - Needs assessment with end users - Literature review - Generation of recommendations for the toolkit - Toolkit development | - Optimism - Environmental Context & Resources | - Knowledge - Social Influences |
| Avery 2016 | - Theory of Planned Behaviour - Social Cognitive Theory - Theoretical Domains Framework - Medical Research Council Framework | - Informing toolkit content and layout - Analyzing results - Guiding the toolkit development process - Guiding questions asked to knowledge users | - Exploratory work (interviews and workshops with end users) - Identification of Active Intervention Ingredients - Assessment of Usability of the Alpha Prototype Intervention - Evaluation to Optimize Intervention Components and Procedures | - Memory - Social/Professional Role/Identity - Beliefs about Consequences - Optimism - Environmental Context & Resources - Social Influences | - Knowledge - Skills - Beliefs about Capabilities - Intentions - Optimism |
| Bolster 2021 | NR | N/A | - Exploratory work (co-creation sessions - Toolkit prototype development (sprint weeks) - Initial toolkit prototype testing - Validation of prototypes with subject experts - Evaluation of toolkit prototypes | - Beliefs about Capabilities | NR |
| Carr 2021 | - Staying Strong Framework | - Informing toolkit content and layout | - Co-designed toolkit content - Reviewed toolkit content - Initial prototype built - Validation of prototype (trial of prototype with panel members) - Dissemination | NR | NR |
| Chock 2011 | NR | N/A | - Initial development of the toolkit - Participant feedback on prototype - Toolkit disseminated to providers - Evaluation of the toolkit at a health clinic | NR | NR |
| Dennett 2022 | - Theoretical Domains Framework | - Guiding questions asked to knowledge users | - Needs assessment with end users (e.g., interviews, workshops) - Pilot evaluation | - Environmental Context & Resources | NR |
| Evans 2007 | - Action Research Framework - Self-regulation Theory | - Informing toolkit content and layout | - Needs assessment with end users (e.g., focus groups) - Toolkit development phase - Analyzing and refining toolkit materials | NR | NR |
| Gale 2024 | - COM-B model - Theoretical Domains Framework | - Analyzing results | - Exploratory work (e.g., focus groups, interviews, discussions with coproduction company) - Evaluation of the usability of the prototype | - Environmental Context & Resources | NR |
| Glowacki 2019 | - Rogers' Diffusion of Innovations Theory - Theoretical Domains Framework | - Informing toolkit content and layout - Analyzing results - Informing intervention strategies | - Literature review & formative interviews - Expert panel meeting - Final toolkit development | - Skills - Beliefs about Consequences - Environmental Context & Resources | - Environmental Context & Resources - Social Influences |
| Goff 2023 | - Self-determination Theory - Principles of andragogy - Bandura's Self-efficacy Theory - Lorig’s Framework of Self-efficacy | - Informing toolkit content and layout | - Exploratory work (e.g., review, discussions with end users) - Toolkit prototype development - Evaluation and iteration of toolkit prototype | - Beliefs about Capabilities - Social/Professional Role/Identity | NR |
| Jones 2021 | - Normalization process theory | - Guiding questions asked to knowledge users | - Development of toolkit prototype - Co-design and review of the tool - Evaluation of the toolkit | - Beliefs about Consequences - Environmental Context & Resources | - Social/Professional Role/Identity |
| Kebbe 2020 | - Social Ecological Model - Social Cognitive Theory | - Informing toolkit content and layout | - Exploratory work (e.g., survey with end users) - Toolkit co-design - Toolkit development and dissemination | - Skills | - Knowledge - Skills - Beliefs about Capabilities - Environmental Context & Resources |
| Levy 2025 | - COM-B model - Theoretical Domains Framework | - Guiding questions asked to knowledge users - Analyzing results | - Exploratory work (e.g., survey with end users) - Co-design of the toolkit | - Knowledge - Environmental Context & Resources - Skills | - Knowledge - Social/Professional Role/Identity - Optimism - Environmental Context & Resources - Skills |
| Mateo 2018 | - Theory of Planned Behavior - 5As Framework - ORBIT model | - Informing toolkit content and layout - Guiding the toolkit development process | - Toolkit development phase - Toolkit evaluation | NR | NR |
| Middleton 2023 | - Knowledge to Action cycle - Authentic Partnership - Behaviour Change Wheel | - Analyzing results - Guiding the toolkit development process - Informing intervention strategies - Guiding knowledge user engagement | - Exploratory work for barriers identification - Identification of target audience and actions, - Iterative development of the toolkit - Usability testing - Evaluation of toolkit impact | - Skills | NR |
| Noël 2018 | NR | N/A | - Exploratory work - Co-designing the toolkit - Evaluation | - Knowledge | - Skills - Memory |
| Osunlana 2015 | - 5As of obesity management framework | - Informing toolkit content and layout | - Needs assessment - Tool co-development - Evaluation | - Skills | - Environmental Context & Resources |
| Sturgiss 2016 | - Knowledge to Action Framework - Normalisation Process Theory | - Analyzing results - Guiding the toolkit development process | - Assessment of barriers to knowledge use - Adapting knowledge to local context - Tailoring the intervention | - Environmental Context & Resources | NR |
| Verwey 2016 | - Transtheoretical model of behaviour change - 5 As model - Theory of Planned Behaviour - Goal-Setting Theory - Self-Determination Theory | - Informing toolkit content and layout | - Exploratory work (literature review and use case) - Interviews with end users and iterative toolkit development - Evauation of the prototype | - Beliefs about Capabilities - Social/Professional Role/Identity - Beliefs about Consequences - Environmental Context & Resources | NR |

N/A: not applicable; NR: not reported
